# CovidArray: A Microarray-Based Assay with High Sensitivity for the Detection of Sars-Cov-2 in Nasopharyngeal Swabs

**DOI:** 10.3390/s21072490

**Published:** 2021-04-03

**Authors:** Francesco Damin, Silvia Galbiati, Stella Gagliardi, Cristina Cereda, Francesca Dragoni, Claudio Fenizia, Valeria Savasi, Laura Sola, Marcella Chiari

**Affiliations:** 1 Istituto di Scienze e Tecnologie Chimiche “Giulio Natta” SCITEC CNR, 20131 Milan, Italy; laura.sola@scitec.cnr.it (L.S.); marcella.chiari@scitec.cnr.it (M.C.); 2 Complications of Diabetes Units, Diabetes Research Institute, IRCCS San Raffaele Scientific Institute, 20132 Milan, Italy; galbiati.silvia@hsr.it; 3 Genomic and Post Genomic Unit, IRCCS Mondino Foundation, 27100 Pavia, Italy; stella.gagliardi@mondino.it (S.G.); cristina.cereda@mondino.it (C.C.); francesca.dragoni@mondino.it (F.D.); 4 Department of Biology and Biotechnology “L. Spallanzani”, University of Pavia, 27100 Pavia, Italy; 5 Department of Pathophysiology and Transplantation, University of Milan, 20122 Milan, Italy; claudio.fenizia@unimi.it; 6 Unit of Obstetrics and Gynecology, L. Sacco Hospital ASST Fatebenefratelli Sacco, 20157 Milan, Italy; valeria.savasi@unimi.it; 7 Department of Biomedical and Clinical Sciences, University of Milan, 20122 Milan, Italy

**Keywords:** SARS-CoV-2, microarray, RT-qPCR, microarray-based assay, Covid-19, molecular diagnostics

## Abstract

A new coronavirus (SARS-CoV-2) caused the current coronavirus disease (Covid-19) epidemic. Reverse transcription-quantitative polymerase chain reaction (RT-qPCR) is used as the gold standard for clinical detection of SARS-CoV-2. Under ideal conditions, RT-qPCR Covid-19 assays have analytical sensitivity and specificity greater than 95%. However, when the sample panel is enlarged including asymptomatic individuals, the sensitivity decreases and false negatives are reported. Moreover, RT-qPCR requires up to 3–6 h with most of the time involved in RNA extraction from swab samples. We introduce CovidArray, a microarray-based assay, to detect SARS-CoV-2 markers N1 and N2 in the nasopharyngeal swabs. The method is based on solid-phase hybridization of fluorescently-labeled amplicons upon RNA extraction and reverse transcription. This approach combines the physical-optical properties of the silicon substrate with the surface chemistry used to coat the substrate to obtain a diagnostic tool of great sensitivity. Furthermore, we used an innovative approach, RNAGEM, to extract and purify viral RNA in less than 15 min. We correctly assigned 12 nasopharyngeal swabs, previously analyzed by RT-qPCR. Thanks to the CovidArray sensitivity we were able to identify a false-negative sample. CovidArray is the first DNA microarray-based assay to detect viral genes in the swabs. Its high sensitivity and the innovative viral RNA extraction by RNAGEM allows the reduction of both the amount of false-negative results and the total analysis time to about 2 h.

## 1. Introduction

In December 2019, an unexplained acute respiratory disease, Covid-19, was first reported in Wuhan, China [1]. It was immediately determined that the disease’s cause was a novel coronavirus named SARS-CoV-2 [2]. Since virus identification and sequencing in early January 2020 [3], the primary approach for detecting viral RNA in respiratory specimens was the reverse transcription-quantitative polymerase chain reaction (RT-qPCR) [4,5]. The RT-qPCR, initially used to confirm symptomatic patients’ diagnosis, was increasingly used to screen asymptomatic contacts and subjects at risk. Several RT-qPCR assays have been developed and recommended by the World Health Organization, the United States Centers for Disease Control and Prevention, the Chinese Center for Disease Control and Prevention, as well as by private companies [6,7]. In the absence of specific therapeutic drugs for Covid-19, it is essential to detect the disease at early stage and immediately isolate the person with Covid-19 from a healthy population. RT-qPCR Covid-19 assays have analytical sensitivity and specificity greater than 95% [8]. However, this number refers to tests validated under ideal conditions with hospital samples containing viral loads higher than those from asymptomatic individuals. When the sample panel is enlarged to include asymptomatic individuals, the sensitivity decreases and false-negative rates between 2% and 33% are reported [8]. In the current emergency, individuals with Covid-19 that are not identified and quarantined represent a transmission vector for a larger amount of the population given the highly contagious nature of the virus. Failures in SARS-CoV-2 detection may be related to multiple preanalytical and analytical factors, such as lack of standardization for specimen collection, poor storage conditions before arrival in the laboratory, and lack of specialized personnel for collection and analysis of the samples. Furthermore, poorly validated assays, contamination during the procedure, insufficient viral specimens, or viral load contribute to results’ uncertainty. The variable viral load along the disease’ incubation period or the presence of mutations and PCR inhibitors also impact the diagnosis [9,10].

In addition, shortage of reagents is also greatly contributing to the spread of the disease by delaying the Covid-19 diagnostics and reducing the number of tests available. Many regions around the world have experienced a shortage of laboratory-based molecular-assay tests. Executing a test requires about 20 different reagents, consumables, and other pieces of equipment. Of those materials, major shortages have been reported in RNA-extraction kits [11]. 

We propose an approach to detect SARS-CoV-2 endowed with high sensitivity, accuracy, and multiplexing capability based on microarray technology, named CovidArray. The method is based on solid-phase hybridization of fluorescently-labeled amplicons upon RNA extraction, and reverse transcription. DNA microarrays have already been used in the genotyping or the detection of pathogens or microorganisms [12,13,14]. However, to the best of our knowledge, there are no examples in the literature of detection of SARS-CoV-2 virus in nasopharyngeal swabs by this technology, that meets the sensitivity and specificity requirements for diagnostic purposes. Essential features of this system, already successfully applied in liquid biopsy and prenatal diagnosis [15,16,17], are: (i) the use of crystalline silicon chips coated by a 100 nm thermally grown silicon dioxide (SiO_2_) layer to enhance fluorescence signals [18], and (ii) the efficient surface chemistry used to bind to the substrate the oligonucleotide capture probes specific to the virus genes [19]. A three-dimensional coating made of N,N-dimethylacrylamide (DMA), N,N-acryloyloxysuccinimide (NAS), and 3-(trimethoxysilyl) propyl methacrylate (MAPS) copolymer (copoly(DMA-NAS-MAPS)), a copolymer known for its high binding capacity and low non-specific adsorption, was used [20]. To validate the microarray-based assay results, we exploited the high sensitivity of the droplet digital PCR (ddPCR) technique. ddPCR, whose limit of detection (LOD) has been shown by several works to be significantly lower than that of the quantitative polymerase chain reaction (qPCR) [21,22], is based on the principles of limited dilution, end-point PCR, and Poisson statistics, with absolute quantification as its heart [23].

In this work, we followed the guidelines of the US Centers for Disease Control and Prevention (CDC) and the CDC 2019-Novel Coronavirus (2019-nCoV) RT-qPCR Diagnostic Panel [24] for the qualitative detection of RNA from SARS-CoV-2 in upper and lower respiratory specimens (such as nasopharyngeal or oropharyngeal swabs). The oligonucleotide primers and probes were selected from two virus nucleocapsid (N1 and N2) gene regions. An additional set of primers/probe to detect the human Ribonuclease P gene (RPP30) as a control of nucleic acid extraction in clinical specimens was also included in the panel. Many available commercial RT-qPCR kits employ a multiplex system capable of detecting 2 or 3 different SARS-CoV-2 targets as well as an internal control. Similarly, the CovidArray assay is able to perform a multiplex detection of the markers N1, N2, and RPP30, but, compared to the commercial RT-qPCR kits, our system has the additional potential to distinguish the SARS-CoV-2 variants or to differentiate it from other viral and bacterial respiratory tract infections simply by adding new primers and capture probes to the same array. 

Another key improvement introduced by CovidArray is the faster analysis time. An important feature of a diagnostic assay during an outbreak is the overall execution time since a fast method would allow expanding the analytical throughput. The introduction of rapid antigen detection (RAD) tests based on immunoassays for qualitative determination of SARS-CoV-2 antigens allows one to obtain a result in about 15–30 min. However, due to their low sensitivity especially for asymptomatic subjects [25], the actual negativity of the sample must in any case be confirmed by the nucleic acid test. Moreover, when they were compared with RT-qPCR, RADs were identified as positive samples containing large amounts of virus but even the most sensitive RAD failed in detecting viral antigens in samples containing small amounts of virus [26]. Thus, rapid tests have the potential to allow earlier detection of those samples testing positive, but the evidence actually is not solid enough to determine how useful they are in clinical practice [27]. The standard methodology for SARS-CoV-2 detection requires from 3 to 6 h to run a test [28] with most of the time involved in RNA extraction from swab samples, with CovidArray the time required is reduced to about 2 h. Many commercial RNA extraction kits such as, for example, the Roche MagNA Pure 96 or the QIAGEN QIAcube kits have been validated for viral RNA extraction purpose [24]. Although the RNA isolation kits are easy to use in automated instruments, it might be necessary to use alternative approaches to extraction to expand the analytical capability in the case of an epidemic. In an attempt to overcome the limits of the standard solid-phase extraction methods many other techniques based, for example, on electrokinetic concentration or isotachophoresis were tried over the years [29,30], but these methods are not still utilized on a large scale. In this work, we used an innovative single-tube approach to extract the viral RNA, by employing RNAGEM, a straightforward temperature-driven enzymatic method to extract RNA, commercially available by MicroGEM (MicroGEM UK Ltd., Southampton, UK). The main advantages of using this extraction methodology are: (i) minimal pipetting steps (manual or automated) leading to less contamination, virtually no loss of RNA and reduced amount of plastic consumables (pipette tips, tubes, etc. also in shortage during this pandemic), (ii) no need of using a harsh chemical which eliminates the washing steps, (iii) no need for further purification of the RNA for accurate RT-qPCR and qPCR analysis and (iv) single-tube workflow that provides purified RNA in 15 min, (v) extraction is conducted using a common laboratory thermocycler allowing to extract up to 96 samples simultaneously, (vi) the manual steps can be automated easily by using any liquid handler. 

Moreover, here we demonstrated that combination of single-tube extraction by RNAGEM with highly sensitive multiplex microarray substrate with optimal properties allows to reduce the number of PCR cycles from 40 to 25 and to lead to an overall increase in accuracy and a reduction in analysis time.

## 2. Materials and Methods

### 2.1. Materials and Reagents

Copoly(DMA-NAS-MAPS) (MCP-4) was obtained by Lucidant Polymers Inc., Sunnyvale, CA, USA. Ammonium sulfate ((NH4)2SO4), ethanolamine and 20× standard saline sodium citrate (SSC) solution (3 M sodium chloride, 0.3 M sodium citrate, pH 7.0), sodium dodecyl sulfate (SDS), were purchased from Sigma Aldrich (St. Louis, MO, USA). All the oligonucleotides were synthesized by Metabion International AG (Steinkirchen, Germany). Their sequences are reported in Supplemental Table S1. Untreated silicon/silicon oxide chips with 100 nm thermal grown oxide (15 × 15 mm^2^) were supplied by SVM, Silicon Valley Microelectronics Inc. (Santa Clara, CA, USA). Chips were pretreated using a HARRICK Plasma Cleaner, PDC-002 (Ithaca, NY, USA) connected to an oxygen line.

Spotting is performed using a SciFLEXARRAYER S12 (Scienion, Berlin, Germany). InnoScan 710 (Innopsys, Carbonne, France) was used to scan the hybridized chips. Data intensities were extracted with the Mapix software and the data analysis was performed for each experiment.

### 2.2. Samples Collection

Five nasopharyngeal swabs have been collected at the Unit of Obstetrics and Gynecology, L. Sacco COVID19-hub Hospital, ASST Fatebenefratelli Sacco, and seven nasopharyngeal swabs have been collected at the IRCCS Mondino Foundation (Pavia). This study was developed on existing samples collected during standard diagnostic tests that were positive to SARS-CoV-2 RNA detection. Subjects participating in the study gave their informed consent (oral or written) for SARS-CoV-2 analysis. The protocol was approved by the local Medical Ethical and Institutional Review Board (Milan, area 1, #154082020). We obtained informed consent from the patients, according to CARE guidelines and in compliance with the Declaration of Helsinki principles.

### 2.3. RNA Extraction and RT-qPCR

Maxwell^®^ RSC Viral Total Nucleic Acid Purification Kit was used to extract RNA from 250 µL of the five nasopharyngeal swabs preservation media (from L. Sacco COVID19-hub Hospital) employing the Maxwell^®^ RSC Instrument (Promega, Fitchburg, WI, USA) while RNAs from 350 µL of the seven nasopharyngeal swabs UTM (IRCCS Mondino Foundation) have been isolated by Magnetic bead method using an automatic nucleic acid purification system (GenePure Pro BIOER) for qPCR testing. 

Dual-labeled TaqMan probes with 5′-6-FAM fluorescent dye and 3′- BHQ-1 quencher for SARS-CoV-2 target sequences N1 was used for the detection of viral RNA. For internal reference control, a pair of primers and TaqMan probe for human Ribonuclease P gene (RPP30), labeled with 5′-HEX fluorescent dye and 3′-BHQ-1 quenchers were used. Primer use has been indicated by US Centers for Disease Control and Prevention [CDC 2019-Novel Coronavirus (2019-nCoV) Real-Time RT-PCR Diagnostic Panel]. 

For reaction mix, 3 µL of extracted RNA and 7 µL of Go-Script One-Step PCR mix (Promega, Madison, WI, USA) have been used for qPCR in CFX96 (BioRad, Richmond, CA) at Sacco Hospital, while 5 µL of extracted RNA, 5 µL of Reliance One-Step RT-qPCR Supermix (BioRad, Richmond, CA), 1 µL of RT enzyme and 4 µL of water have been used at Mondino Foundation, depending on the protocol used. Cycling conditions were 50 °C for 10 min, 95 °C for 3 min, followed by 40 cycles of amplification (95 °C for 10 s and 60 °C for 30 s). qPCR analysis has been considered valid in all samples in which RPP30 gene has been detected. Positive samples were determinate by Cycle threshold (Ct) of N1 and N2 gene minor of 40.

### 2.4. RNA Extraction for CovidArray Analysis

The viral RNA was extracted by mixing 89.5 µL of the inactivated universal transport medium (UTM) (70 °C for 1 h) containing a nasopharyngeal swab with 0.5 µL of RNAGEM enzyme (MicroGEM UK Ltd., Southampton) and 10 µL of 10× Blue Buffer. Subsequently, the extraction was conducted by incubating the reaction mix in a thermocycler at 75 °C for 10 min and 95 °C for 5 min.

### 2.5. Reverse Transcription

8 µL of the RNA extracted were converted in cDNA using the SuperScript™ First-Strand Synthesis System (III) kit from ThermoFisher. The reverse transcription step was carried out according to the manufacturer’s instructions. The obtained cDNA was diluted 1:20.

### 2.6. PCR Conditions for Microarray

The N1, N2, and RPP30 sequences were amplified using the 5′-biotin forward and 5′-Cy3 labeled reverse primers reported in Table S1.

The PCRs were performed in 20 µL of reactions containing 9 µL of cDNA previously diluted 1:20, 200 µM deoxynucleotide triphosphates, 10 mM Tris–HCl (pH 8.3), 50 mM KCl, 1.5 mM MgCl2, 1 U of DNA polymerase (FastStart Taq, Roche) and 10 pmoles of each primer. 

Cycling conditions were as follows: 95 °C for 5 min; 25 cycles at 95 °C for 30 s, 60 °C for 30 s, 72 °C for 30 s and finally 72 °C for 10 min.

In addition to the single amplification, we optimized a triplex PCR amplification in which the primers used for the N1, N2, and RPP30 amplification were mixed in the same PCR mixture.

The triplex PCR was performed in 20 µL of reactions containing 9 µL of diluted cDNA, 10 pmoles of each primer, and 4 µL of 5× HOT FIREPol Blend Master Mix Ready to Load (Solis BioDyne).

Cycling conditions were as follows: 95 °C for 12 min; 25 cycles at 95 °C for 20 s, 60 °C for 30 s, 72 °C for 30 s, and finally 72 °C for 7 min.

### 2.7. Microarray Preparation

We selected three oligonucleotide sequences from the US CDC 2019-Novel Coronavirus (2019-nCoV) Real-Time RT-qPCR Diagnostic Panel corresponding to the two specific probes for the regions N1 and N2 of the virus nucleocapsid gene and to the human RPP30 gene. In addition, an oligonucleotide probe, not correlated to any viral or human sequences, was selected as negative hybridization control. Capture and control probes (reported in Table S1), amino-modified at the 5′ end, were dissolved in the printing buffer (150 mM sodium phosphate pH 8.5, 0.01% Sucrose monolaurate) to a concentration of 10 μM and printed by a piezoelectric spotter, SciFLEX ARRAYER S12 (Scienion, Berlin, Germany) on silicon/silicon oxide slides coated with MCP-4 according to the protocol provided by the manufacturer. The spotting was performed at 20 °C in an atmosphere of 60% humidity.

After the spotting step the chips were incubated overnight, and all residual reactive groups of the coating polymer were blocked as previously described [31].

### 2.8. Microarray Hybridization and Detection

The products of the PCR reactions were heated at 95 °C for 5 min to denature the DNA double-strand. The solution was quickly centrifuged and chilled on ice for 1 min then it was spread onto the microarray. A cover slip (large enough to cover the entire spotted surface) was carefully placed on the microarray to avoid any bubble capture. The slides were incubated in a sealed humid hybridization chamber at room temperature for 15 min. The hybridized silicon chips were then removed from the hybridization chamber and soaked briefly in 4× SSC buffer to remove the cover slips. Finally, the chips were washed at room temperature with 0.2× SSC for 1 min and 0.1× SSC for 1 min and then dried with a nitrogen flow. 

The hybridized silicon chips were scanned with InnoScan 710 (Innopsys, Carbonne, France). A green laser (λ_ex_ 532 nm) for the Cy3 dye was applied. The photomultiplier (PMT) tube gain and the laser power changed between different experiments. 16-bit TIFF images were analyzed at 5 µm resolution. Data intensities were extracted with the Mapix software and the data analysis was performed for each experiment.

### 2.9. Droplet Digital PCR (ddPCR)

We employed the QX100™ Droplet Digital™ PCR System (Bio-Rad Laboratories, Hercules, CA, USA). 9 μL of cDNA previously diluted 1:20 were mixed with primers and fluorophore-labeled commercial probes (2019-nCoV CDC EUA Authorized qPCR Probe Assay primer/probe mix, Integrated DNA Technologies) specific for the amplification of N1, N2 and RPP30 genes as previously reported. The volume of the PCR mix was 20 µL including 10 µL of ddPCR™ Supermix for Probes (No dUTP) and 1 µL of primers/probe. The droplet emulsion was thermally cycled on C1000 Touch Thermal Cycler (Bio-Rad) instrument. Cycling conditions were 95 °C for 5 min, followed by 40 cycles of amplification (94 °C for 30 s and 60 °C for 1 min), ending with 98 °C for 10 min, according to the manufacturer’s protocol. The copies of the target gene were calculated automatically by the QuantaSoft™ software version 1.7.4 (Bio-Rad).

### 2.10. 2019-CoV Plasmid Controls

Plasmid controls contain the complete nucleocapsid gene from 2019-nCoV virus were provided by Integrated DNA Technologies and delivered at a concentration of 200,000 copies/µL in IDTE pH 8.0.

## 3. Results and Discussion

### 3.1. Detection of SARS-CoV-2 Nucleic Acid from Clinical Nasopharyngeal Swab Samples with CovidArray

**Figure 1:**
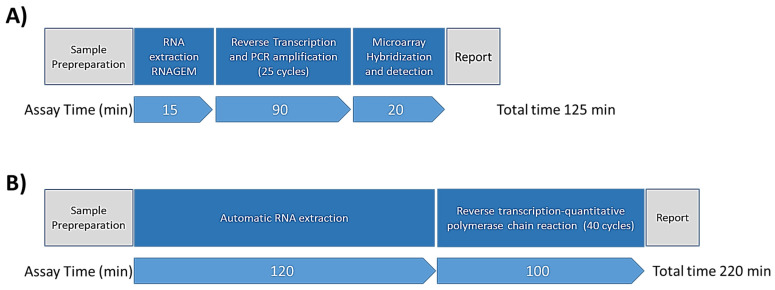
Flowchart and timeline of the SARS-CoV-2 diagnostic workflow for CovidArray (**A**) and standard reverse transcription-quantitative polymerase chain reaction (RT-qPCR) assay [28] (**B**).

An oligonucleotide microarray targeting two regions (N1 and N2) of the Sars-CoV-2 nucleocapsid, and the human RPP30, was developed (Figure 1A).

To interpret the results, the indications of the US CDC qPCR test were followed. In this assay, the fluorescence of capture spots indicates positivity. Briefly, a specimen is considered positive for SARS-CoV-2 if the two SARS-CoV-2 markers (N1, N2) produce a fluorescence signal that exceeds more than 3 times the standard deviation, the signal of the no-template control (NTC). On the contrary, a specimen is considered negative if the SARS-CoV-2 markers (N1, N2) show a signal non-discernible from that of the NTC. The RPP30 gene, in a positive sample, may or may not be positive. It is possible that some samples may fail to exhibit RPP30 fluorescence due to low cell numbers in the original clinical sample. A negative RPP30 signal does not preclude the presence of Sars-CoV-2 virus RNA in a clinical specimen. On the other hand, the absence of the RPP30 signal in a negative specimen makes the result invalid because the presence of an RPP30 signal in a sample negative for N1 and N2 confirms the correct extraction of RNA. 

In this work, to greatly speed up the test, the viral RNA was extracted from the nasopharyngeal swabs (stored at −80 °C) using RNAGEM kit. RNA extraction by single-step reagent isolation methods may leave behind contaminants that reduce amplification efficiency. However, the inhibition that, in standard media, would hamper the RT-PCR detection does not affect sensitivity of CovidArray assay thanks to the increased sensitivity of this microarray approach. To optimize the assay and further reduce the analysis time, the transcripts were amplified at a different number of cycles (data not shown). The optimization showed that the array is able to detect, after only 25 cycles, amplicons that are detectable at a higher number of cycles with standard RT-qPCR. A decrease of the number of cycles to 25 led to reduction of the analysis time (1h of reaction). Figure 1 shows a comparison between the workflows of the CovidArray platform and the standard RT-qPCR method. The limiting step of the standard workflow is the RNA extraction, which is overcome, in our workflow, by the use of the RNAGEM enzyme.

Firstly, it was demonstrated the correct assignment of samples previously assayed in the laboratory of Immunology at University of Milan (L. Sacco Hospital) with standard qPCR technology. In particular, five nasopharyngeal swabs (S1–S5) were analyzed, four of which positive for the target N1 and N2 by RT-qPCR and one negative for both (S3). The four positive samples presented different viral loads resulting in different threshold cycles (Ct) in qPCR. In particular, for N1, the sample S1 crossed the threshold line at 20.00 cycles due to its high viral load; also sample S2 has a high viral load (22.00 cycles) while S4 and S5 have lower viral load and are detected at 38 and 36 cycles respectively (Table 1).

**Figure 2:**
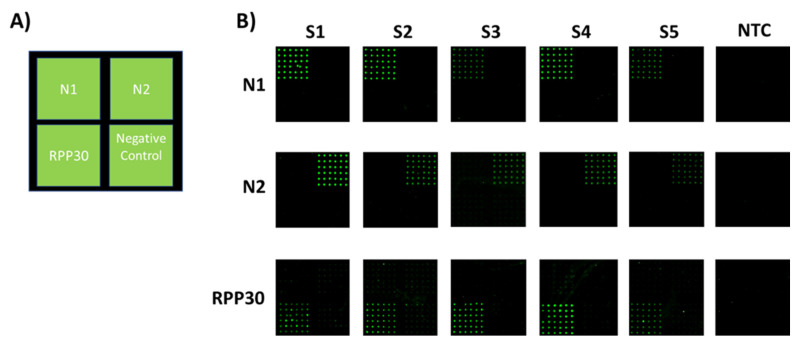
Analysis of 5 nasopharyngeal swabs by CovidArray. (**A**) Spotting schema of the CovidArray. Silicon chips coated with MCP-4 are used as substrates for the covalent attachment of amino-modified capture probe oligonucleotides arrayed at discrete locations. Each position in the grid identifies an individual capture probe address corresponding to nucleocapsid1 and 2 regions (N1 and N2), human Ribonuclease P gene (RPP30), and negative control (not correlated oligonucleotide probe). (**B**) Cy3 fluorescence images of eighteen different silicon chips. Each robotically spotted array is hybridized with an individual single-strand Cy3-labelled polymerase chain reaction (PCR) products corresponding to (from top to bottom) N1, N2, RPP30 amplicons of five nasopharyngeal samples (S1–S5) and with the No Template Control (NTC). Laser Power: Low; PMT: 5%.

Figure 2B shows the results of the microarray analysis of the swab samples and the control NTCs for the SARS-CoV-2 markers (N1 and N2) and for the RPP30 positive control.

**Figure 3:**
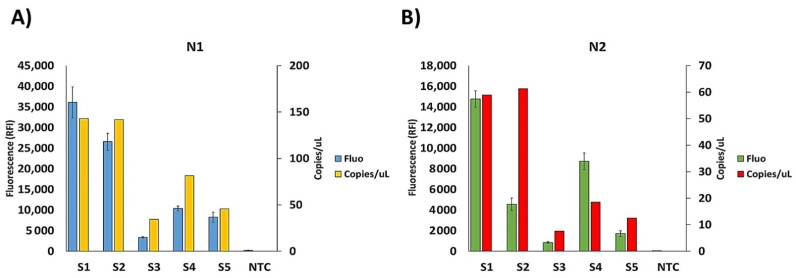
Plots of the relative fluorescence intensity (blue and green bars) of the images in Figure 2B, and the copies number/μL of the corresponding droplet digital PCR (ddPCR) (yellow and red bars), for the N1 (**A**) and N2 (**B**) markers. The blue and green bars are the average of the relative fluorescence intensity (RFI) of the 36 spots (6 × 6 subarrays) of each capture probe subarrays. The error bars are the standard deviations of the fluorescence intensity of the subarray.

Amplicons of N1, N2, and RPP30 were separately incubated on microarray chips. Three different silicon substrates were used to analyze one swab sample. As shown in Figure 2B, the fluorescence signal appears only at the location where the immobilized capture probe is complementary to the labeled PCR with no cross-hybridization and a good reproducibility from spot to spot. The absence of fluorescence in the NTC subarrays is essential as it allows discriminating low-signal samples from background signals. The same samples were also assayed by ddPCR. Indeed, one of our samples (S3) from a patient negative according to the RT-qPCR assay, but with symptoms attributable to Covid-19, was found to be positive by CovidArray in agreement with ddPCR (N1 = 34.5 copies/µL; N2 = 7.6 copies/µL). CovidArray matches the sensitivity of ddPCR (Figure 3). 

The sensitivity increase of the proposed method reduces the number of false-negative results. It also shortens the analysis time by reducing the number of PCR cycles required to detect a positivity. The fluorescence signal intensity of the spots in CovidArray correlates well with the viral load of the five samples with the higher fluorescence corresponding to the samples with the highest viral load. 

The CovidArray was further validated with seven nasopharyngeal swabs from IRCCS Mondino Foundation (Pavia), previously subjected to solid-phase extraction and RT-qPCR as reported in the experimental/materials and methods section. A 100% agreement between the two methods was found (Table 2). The fluorescence images for samples B243, N053, and NTC are shown in Figure S1.

### 3.2. Detection Limit of CovidArray

Since the new SARS CoV-2 emerged, researchers have struggled to develop highly sensitive molecular techniques to diagnose positive Covid-19 subjects effectively. According to the WHO and the Centre for Disease Control and Prevention (CDC), the gold standard for the diagnosis is qPCR. However, many studies have highlighted the presence of false-negative results in RT-qPCR [32,33]. Therefore, it is worthy to build up novel robust methodologies that ensure high sensitivity useful not only for diagnostic purposes but also for the follow-up of patients and for monitoring of the viral load. To evaluate our method’s sensitivity, serial dilutions of linear DNA standard 2019-CoV Plasmid Control were tested using primer sets targeting N1 and N2 regions. The plasmid DNA was diluted to 50, 25, 5, 2.5, 0.5, 0.25, 0.05 copies/μL prior to undergo 25 cycles of PCR. To build the calibration curves for the two viral regions, the capture probes in 36 replicates (6 × 6 subarrays) were spotted on different coated silicon chips (one chip for each plasmid concentration). Samples with decreasing concentration of plasmid DNA were amplified, denatured, and finally hybridized for 15 min at room temperature.

**Figure 4:**
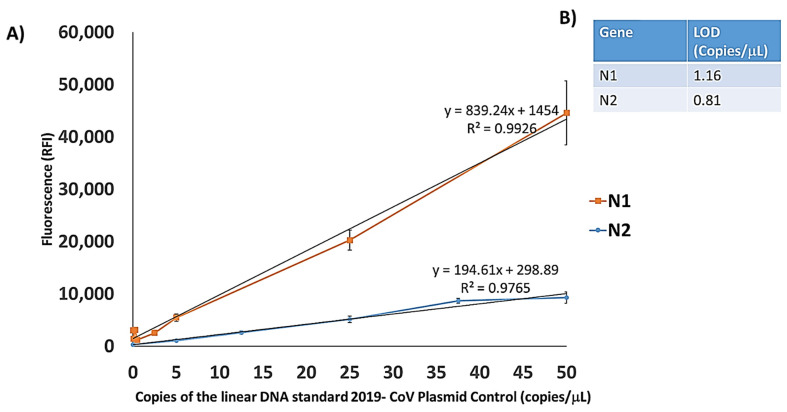
CovidArray Limit of detection. (**A**) Plot of the relative fluorescence intensity (RFI) of the N1 and N2 signals. The points, calculated as the average of the intensity of 36 spots, correspond to the number of copies/μL of linear DNA standard 2019-CoV Plasmid Control. The value at point 0 represents the relative fluorescence intensity of the background with no 2019-CoV Plasmid Control. The error bars are the standard deviations of the fluorescence intensity of each chip. The equations of the trend lines are utilized to extrapolate the limit of detection (LOD) for the assay. (**B**) The extrapolated limits of detection for the N1 and N2 marker.

The value of fluorescence intensity detected for each of the seven plasmid concentrations together with the background fluorescence of the control sample with no plasmid DNA was plotted versus the number of copies of the plasmid per μL in the starting solution. Figure 4A shows the calibration curves for the region N1 and N2, respectively.

The LODs (lowest concentration of detectable plasmid DNA) extrapolated for each marker are reported in Figure 4B. The determination of LOD is based on the equation: 3.3 σ/s where s is the slope of calibration curve and σ is the standard deviation of fluorescence background in the control sample. The LODs found with this system are 1.16 copies/μL for the N1 and 0.81 copies/μL for the N2, respectively. These LODs are comparable with those declared by the various manufacturers of kits for qPCR with the difference that the number of standard amplification cycles for those methods is 40 while, in our approach only 25 cycles are sufficient to detect the target genes.

### 3.3. Multiplex Capability of the CovidArray

**Figure 5:**
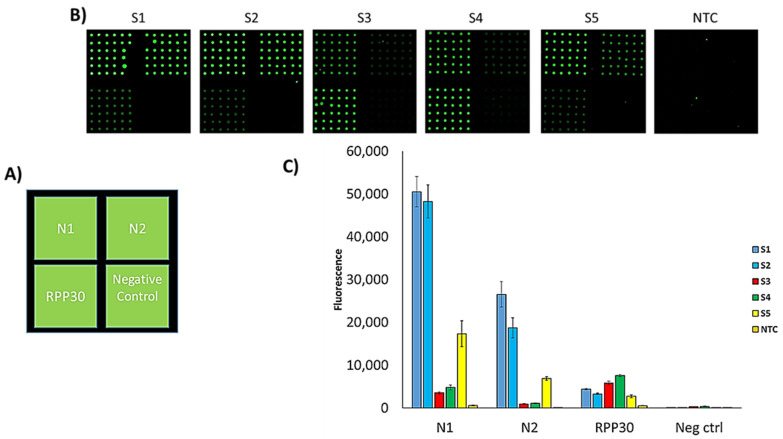
Multiplex analysis of 5 nasopharyngeal swabs by CovidArray. (**A**) Spotting schema of the CovidArray. (**B**) Cy3 fluorescence images of six silicon chips. Each CovidArray is hybridized with a triplex Cy3-labelled PCR specific for N1, N2, RPP30 gene from the samples S1-S5 or with a No Template Control (NTC). (**C**) The plot of the relative fluorescence intensity of the silicon chips shown in panel B. Laser Power: Low; PMT: 5%. All the bars are the average of the intensity of the 36 spots (6 × 6 subarrays) of each capture probe subarrays. The error bars are the standard deviations of the fluorescence intensity of each subarray.

One of the peculiar features of microarray technology is its multiplexing capability. Different genes can be revealed in a single hybridization assay by spotting onto the microarray substrate different capture probes specific to the target. In this work, we exploited the multiplexing capability of the CovidArray to detect the presence of the N1, N2 markers of SARS-CoV-2 and the RPP30 control gene on a single silicon chip in a single hybridization assay. We performed a triplex PCR, amplifying simultaneously the genes N1, N2, and RPP30 using the cDNA produced by reverse transcription of the RNA extracted from the same 5 nasopharyngeal swabs as reported in the “Materials and Method” section. The triplex PCR was hybridized with the probes spotted on the same substrate. The simultaneous appearance of fluorescence signals on the subarrays corresponding to the N1 and N2 regions confirmed the positivity of the sample. The fluorescence signal of the RPP30 subarray was also detectable. In Figure 5B five triplex PCR’ hybridization results corresponding to the S1–S5 samples are shown. The NTC does not show significant fluorescence. In Figure 5C the histogram of fluorescence intensity indicates that the samples S1 and S2 have a higher fluorescence intensity confirming the higher viral load detected by the single-PCR CovidArray. Sample S3 which was considered negative by the RT-qPCR technique shows a weaker but detectable signal in agreement with ddPCR and single-PCR CovidArray.

## 4. Conclusions

In summary, we describe a novel microarray platform, CovidArray, for the specific and sensitive detection of SARS-CoV-2 in nasopharyngeal swabs. This approach combines the physical-optical properties of the silicon substrate with the surface chemistry used to bind to the substrate the oligonucleotide capture probes specific to the virus’ genes to obtain a diagnostic tool of great sensitivity. In agreement with ddPCR, we correctly assigned 12 nasopharyngeal swabs of different origins. Thanks to the lower limit of detection of CovidArray, we identified a false-negative sample. Another feature of our system, also due to the high sensitivity of the CovidArray, is the decrease of the number of PCR cycles required to detect the viral markers which, in turn, leads to a significant reduction of the analysis time. A further contribution to speeding up the diagnostic assay is the use of an alternative method for extracting the viral RNA from clinical samples. 

In this work, we used an innovative approach, RNAGEM, commercially available by MicroGEM, to extract and purify viral RNA in less than 15 min. The total time required for the molecular test can thus range from about 3–6 h of a standard process to about 2 h with the CovidArray method. Moreover, RNAGEM provides an alternative to commercial RNA extraction kits that may undergo a shortage due to their massive use during the pandemic. Furthermore, in this work we have exploited the multiplexing capability of the microarray technology, to detect the presence of viral markers and the control sequence in a single assay.

The drawback of the current platform is the high degree of manual work required to perform the analysis. Our current throughput is still far from that of RT-qPCR. The analytical capability of our approach is only 16 samples at a time. However, there are no conceptual obstacles to integrating the assay into an automatic platform. The whole workflow, from viral RNA extraction to detection can be performed in a microtiter plate equipped with a microplate fluorescence reader.

Finally, it is worth noticing the versatility of this approach. In fact, CovidArray could potentially allow differentiating SARS-CoV-2 from other viral and bacterial respiratory tract infections by merely adding new primers and capture probes to the same array, becoming a promising diagnostic tool suitable for routine diagnosis of a wide range of respiratory diseases.

## Figures and Tables

**Table 1:** RT-qPCR for N1 and N2.

Nasopharyngeal Swabs	N1 qPCR (Ct) ^1^	N2 qPCR (Ct) ^1^
S1	20.61	20.45
S2	21.83	29.31
S3	N/A ^2^	N/A ^2^
S4	38.49	N/A ^2^
S5	35.86	N/A ^2^

^1^ Cycle Threshold. ^2^ not applicable.

**Table 2:** Correspondence between RT-qPCR Ct and CovidArray.

Sample Id	N1 qPCR (Ct) ^1^	N2 qPCR (Ct) ^1^	CovidArray
N017	34.66	39.35	POSITIVE
N053	N/Ab	N/Ab	NEGATIVE
B001	34.74	35.16	POSITIVE
N141	14.48	12.83	POSITIVE
N051	N/A ^2^	N/A ^2^	NEGATIVE
B210	33.24	42.5	POSITIVE
B243	25.82	39.29	POSITIVE

^1^ Cycle Threshold. ^2^ not applicable.

## Data Availability

Data is contained within the article or supplementary material.

## Supplementary Materials

The following are available online at https://www.mdpi.com/article/10.3390/s21072490/s1, Table S1: Assay primer/probe sequences, Figure S1: Cy3 fluorescence image of the CovidArray analysis of sample B243 and N053.

## References

[1] Zhu N., Zhang D., Wang W., Li X., Yang B., Song J., Zhao X., Huang B., Shi W., Lu R. (2020). A Novel Coronavirus from Patients with Pneumonia in China, 2019. N. Engl. J. Med..

[2] Gorbalenya A.E., Baker S.C., Baric R.S., de Groot R.J., Drosten C., Gulyaeva A.A., Haagmans B.L., Lauber C., Leontovich A.M., Neuman B.W. (2020). The species Severe acute respiratory syndrome-related coronavirus: Classifying 2019-nCoV and naming it SARS-CoV-2. Nat. Microbiol..

[3] Zhang Y.Z. Novel 2019 Coronavirus Genome. Virological. http://virological.org/t/novel-2019-coronavirus-genome/319.

[4] World Health Organization Laboratory Testing for 2019 Novel Coronavirus (2019-nCoV) in Suspected Human Cases. https://apps.who.int/iris/handle/10665/330676.

[5] General Office of the National Health and Health Commission Office of the NA of TCM Diagnosis and Treatment of Pneumonitis with a New Type of Coronavirus Infection (Trial Version 7). https://www.who.int/docs/default-source/wpro---documents/countries/china/covid-19-briefing-nhc/1-clinical-protocols-for-the-diagnosis-and-treatment-of-covid-19-v7.pdf?sfvrsn=c6cbfba4_2.

[6] National Medical Products Administration New Novel Coronavirus Detection Products Approved by the National Medical Products Administration. http://www.nmpa.gov.cn/WS04/CL2056/375802.html.

[7] United States Food and Drug Administration https://www.fda.gov/medical-devices/emergency-use-authorizations-medical-devices/coronavirus-disease-2019-covid-19-emergency-use-authorizations-medical-devices.

[8] Arevalo-Rodriguez I., Buitrago-Garcia D., Simancas-Racines D., Zambrano-Achig P., Del Campo R., Ciapponi A., Sued O., Martinez-García L., Rutjes A.W., Low N. (2020). False-negative results of initial RT-QPCR assays for COVID-19: A systematic review. PLoS ONE.

[9] Lippi G., Simundic A.M., Plebani M. (2020). Potential preanalytical and analytical vulnerabilities in the laboratory diagnosis of coronavirus disease 2019 (COVID-19). Clin. Chem. Lab. Med..

[10] Li D., Wang D., Dong J., Wang N., Huang H., Xu H., Xia C. (2020). False-Negative Results of Real-Time Reverse-Transcriptase Polymerase Chain Reaction for Severe Acute Respiratory Syndrome Coronavirus 2: Role of Deep-Learning-Based CT Diagnosis and Insights from Two Cases. Korean J. Radiol..

[11] McKinsey and Company COVID-19: Overcoming Supply Shortages for Diagnostic Testing. http://www.mckinsey.com/industries/pharmaceuticals-and-medical-products/our-insights/covid-19-overcoming-supply-shortages-for-diagnostic-testing?cid=eml-web.

[12] Palka-Santini M., Cleven B.E., Eichinger L., Krönke M., Krut O. (2009). Large scale multiplex PCR improves pathogen detection by DNA microarrays. BMC Microbiol..

[13] Mou X., Ali Z., Li B., Li T., Yi H., Dong H., He N., Deng Y., Zeng X. (2016). Multiple genotyping based on multiplex PCR and microarray. Chin. Chem. Lett..

[14] Zheng Z., Wu Y., Yu X., Shang S. (2008). DNA Microarray Technology for Simultaneous Detection and Species Identification of Seven Human Herpes Viruses. J. Med. Virol..

[15] Damin F., Galbiati S., Soriani N., Burgio V., Ronzoni M., Ferrari M., Chiari M. (2018). Analysis of KRAS, NRAS and BRAF mutational profile by combination of in-tube hybridization and universal tag-microarray in tumor tissue and plasma of colorectal cancer patients. PLoS ONE.

[16] Galbiati S., Damin F., Ferraro L., Soriani N., Burgio V., Ronzoni M., Gianni L., Ferrari M., Chiari M. (2019). Microarray approach combined with ddPCR: An useful pipeline for the detection and quantification of circulating tumor DNA mutations. Cells.

[17] Galbiati S., Monguzzi A., Damin F., Soriani N., Passiu M., Castellani C., Natacci F., Curcio C., Seia M., Lalatta F. (2016). COLD-PCR and microarray: Two independent highly sensitive approaches allowing the identification of fetal paternally inherited mutations in maternal plasma. J. Med. Genet..

[18] Ozkumur E., Yalcin A., Cretich M., Lopez C.A., Bergstein D.A., Goldberg B.B. (2009). Quantification of DNA and protein adsorption by optical phase shift. Biosens. Bioelectron..

[19] Cretich M., di Carlo G., Longhi R., Gotti C., Spinella N., Coffa S., Galati C., Renna L., Chiari M. (2009). High sensitivity protein assays on microarray silicon slides. Anal. Chem..

[20] Pirri G., Damin F., Chiari M., Bontempi E., Depero L.E. (2004). Characterization of a polymeric adsorbed coating for DNA microarray glass slides. Anal. Chem..

[21] Suo T., Liu X., Feng J., Guo M., Hu W., Guo D., Ullah H., Yang Y., Zhang Q., Wang X. (2020). ddPCR: A more accurate tool for SARS-CoV-2 detection in low viral load specimens. Emerg. Microbes. Infect..

[22] Falzone L., Musso N., Gattuso G., Bongiorno D., Palermo C.I., Scalia G., Libra M., Stefani S. (2020). Sensitivity assessment of droplet digital PCR for SARS-CoV-2 detection. Int. J. Mol. Med..

[23] Vogelstein B., Kinzler K.W. (1999). Digital PCR. Proc. Natl. Acad. Sci. USA.

[24] Centers for Disease Control and Prevention (CDC) CDC 2019-Novel Coronavirus (2019-nCoV) Real-Time RT-QPCR Diagnostic Panel. https://www.fda.gov/media/134922/download.

[25] Mak G.C.K., Cheng P.K.C., Lau S.S.Y., Wong K.K.Y., Lau C.S., Lam E.T.K., Chan R.C.W., Tsang D.N.C. (2020). Evaluation of rapid antigen test for detection of SARS-CoV-2 virus. J. Clin. Virol..

[26] Yamayoshi S., Sakai-Tagawa Y., Koga M., Akasaka O., Nakachi I., Koh H., Maeda K., Adachi E., Saito M., Nagai H. (2020). Comparison of Rapid Antigen Tests for COVID-19. Viruses.

[27] Dinnes J., Deeks J.J., Adriano A., Berhane S., Davenport C., Dittrich S., Emperador D., Takwoingi Y., Cunningham J., Beese S. (2020). COVID-19 Diagnostic Test Accuracy Group. Rapid, point-of-care antigen and molecular-based tests for diagnosis of SARS-CoV-2 infection. Cochrane Database Syst. Rev..

[28] Konrad R., Eberle U., Dangel A., Treis B., Berger A., Bengs K., Fingerle V., Liebl B., Ackermann N., Sing A. (2020). Rapid establishment of laboratory diagnostics for the novel coronavirus SARS-CoV-2 in Bavaria, Germany, February 2020. Eurosurveillance.

[29] Han C.M., Catoe D., Munro S.A., Khnouf R., Snyder M.P., Santiago J.G., Salit M.L., Cenik C. (2019). Simultaneous RNA purification and size selection using on-chip isotachophoresis with an ionic spacer. Lab Chip.

[30] Ouyang W., Han J. (2020). One-Step Nucleic Acid Purification and Noise-Resistant Polymerase Chain Reaction by Electrokinetic Concentration for Ultralow-Abundance Nucleic Acid Detection. Angew. Chem. Int. Edit..

[31] Damin F., Galbiati S., Ferrari M., Chiari M. (2016). DNA microarray-based solid-phase PCR on copoly (DMA–NAS–MAPS) silicon coated slides: An example of relevant clinical application. Biosens. Bioelectron..

[32] Wang X., Yao H., Xu X., Zhang P., Zhang M., Shao J., Xiao Y., Wang H. (2020). Limits of detection of six approved RT-QPCR kits for the novel SARS-coronavirus-2 (SARS CoV-2). Clin. Chem..

[33] Tahamtan A., Ardebili A. (2020). Real-time RT-QPCR in COVID 19 detection: Issues affecting the results. Expert. Rev. Mol. Diagn..

