# Supplementary material for: CovidArray: A Microarray-Based Assay with High Sensitivity for the Detection of Sars-Cov-2 in Nasopharyngeal Swabs
Source: Sensors (Basel). 2021 Apr 3;21(7):2490. doi: 10.3390/s21072490 (PMC8038375; doi:10.3390/s21072490)
Supplement: Supplementary file 1 [file sensors-21-02490-s001.pdf]

sensors

MDPI

Supplementary Materials

CovidArray: A Microarray-Based Assay with High Sensitivity for the Detection of Sars-Cov-2 in Nasopharyngeal Swabs

Francesco Damin [1,*,†], Silvia Galbiati [2,†], Stella Gagliardi [3], Cristina Cereda [3], Francesca Dragoni [3,4], Claudio Fenizia [5], Valeria Savasi [6,7], Laura Sola [1] and Marcella Chiari [1]

[1] Istituto di Scienze e Tecnologie Chimiche "Giulio Natta" SCITEC CNR, 20131 Milan, Italy; laura.sola@scitec.cnr.it (L.S.); marcella.chiari@scitec.cnr.it (M.C.)
[2] Complications of Diabetes Units, Diabetes Research Institute, IRCCS San Raffaele Scientific Institute, 20132 Milan, Italy; galbiati.silvia@hsr.it
[3] Genomic and Post Genomic Unit, IRCCS Mondino Foundation, 27100 Pavia, Italy; stella.gagliardi@mondino.it (S.G.); cristina.cereda@mondino.it (C.C.); francesca.dragoni@mondino.it (F.D.)
[4] Department of Biology and Biotechnology "L. Spallanzani", University of Pavia, 27100 Pavia, Italy
[5] Department of Pathophysiology and Transplantation, University of Milan, 20122 Milan, Italy; claudio.fenizia@unimi.it
[6] Unit of Obstetrics and Gynecology, L. Sacco Hospital ASST Fatebenefratelli Sacco, 20157 Milan, Italy; valeria.savasi@unimi.it
[7] Department of Biomedical and Clinical Sciences, University of Milan, 20122 Milan, Italy
* Correspondence: francesco.damin@scitec.cnr.it
† These authors contribute equally to this work.

Citation: Damin, F.; Galbiati, S.; Gagliardi, S.; Cereda, C.; Dragoni, F.; Fenizia, C.; Savasi, V.; Sola, L.; Chiari, M. CovidArray: A Microarray-Based Assay with High Sensitivity for the Detection of Sars-Cov-2 in Nasopharyngeal Swabs. *Sensors* **2021**, *21*, 2490. https://doi.org/10.3390/s21072490

Received: 1 March 2021
Accepted: 31 March 2021
Published: 3 April 2021

Publisher's Note: MDPI stays neutral with regard to jurisdictional claims in published maps and institutional affiliations.

Copyright: © 2021 by the authors. Licensee MDPI, Basel, Switzerland. This article is an open access article distributed under the terms and conditions of the Creative Commons Attribution (CC BY) license (http://creativecommons.org/licenses/by/4.0/).

Table S1. Assay primer/probe sequences.

Assay	Description		Oligonucleotide Sequence	Amplicon Size (bp)
N1	Nucleocapside gene	For	5′-GACCCCAAAATCAGCGAAAT-3′	73
		Rev[1]	5′-TCTGGTTACTGCCAGTTGAATCTG-3′	
		Probe[2]	5′-ACCCCGCATTACGTTTGGTGGACC-3′	
N2	Nucleocapside gene	For	5′-TTACAAACATTGGCCGCAAA-3′	67
		Rev[1]	5′-GCGCGACATTCCGAAGAA-3′	
		Probe[2]	5′- ACAATTTGCCCCCAGCGCTTCAG-3′	
RPP30	Ribonuclease P gene	For	5′-AGATTTGGACCTGCGAGCG-3′	65
		Rev[1]	5′-GAGCGGCTGTCTCCACAAGT-3′	
		Probe[2]	5′-TTCTGACCTGAAGGCTCTGCGCG-3′	
Negative control	Hybridization control	Probe[2]	5′-AGGGCTCTATTCAGCGTATT-3′	

All the sequences are from the US CDC 2019-Novel Coronavirus (2019-nCoV) Real-Time RT-QPCR Diagnostic Panel. [1]The Reverse primers are labeled with Cyanine 3 in 5′-end. [2]The spotted capture probes are amino modified in 5′-end.

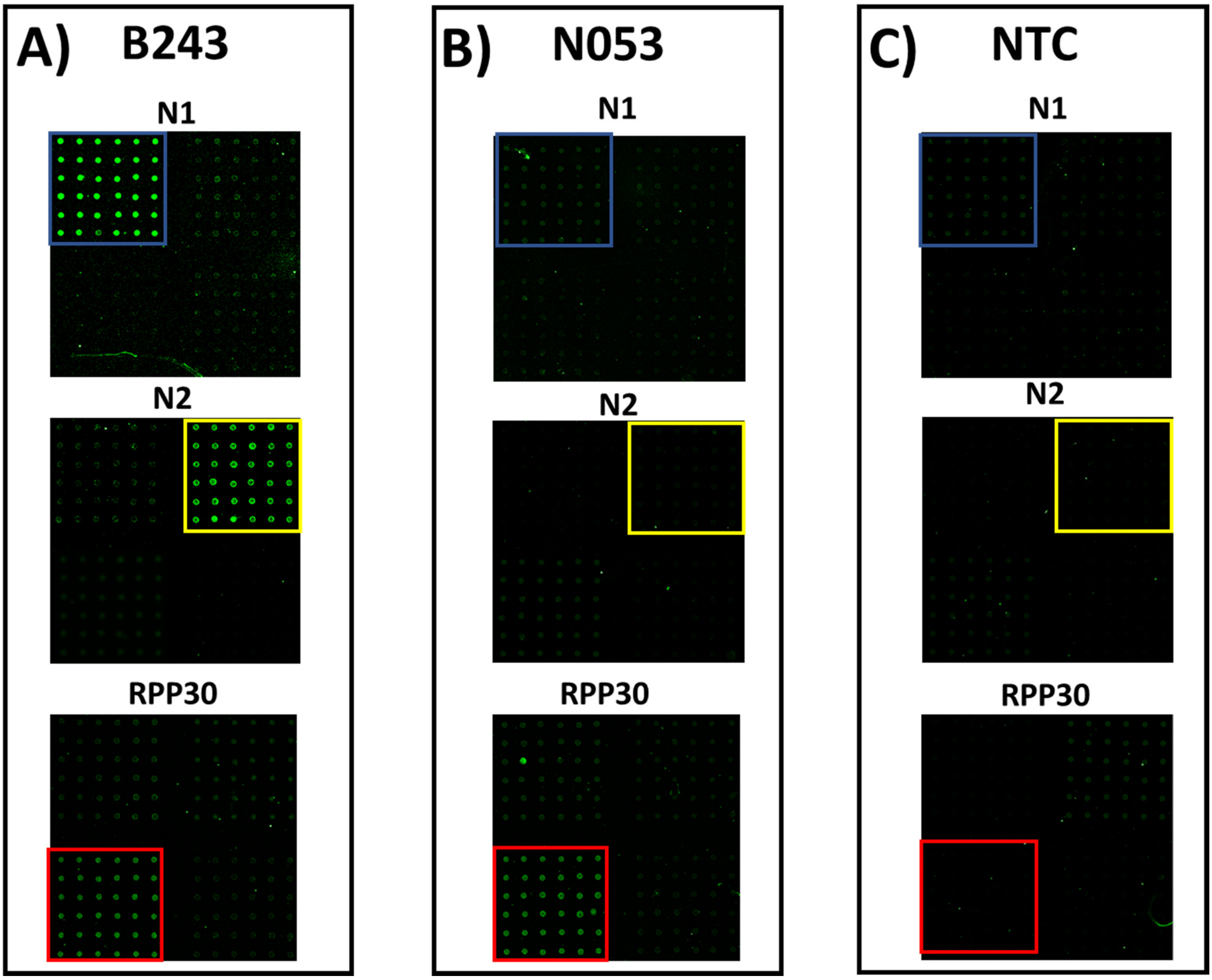

Figure S1. CovidArray analysis of two nasopharyngeal swabs. (**A**) Cy3 fluorescence image of the CovidArray analysis of sample B243. N1, N2 and RPP30 subarrays are highlighted in blue, yellow, and red, respectively. (**B**) Cy3 fluorescence images of the CovidArray analysis of sample N053. N1, N2 and RPP30 subarrays are highlighted in blue, yellow, and red, respectively. (**C**) Cy3 fluorescence image of the CovidArray analysis of the No Template Control (NTC). N1, N2 and RPP30 subarrays are highlighted in blue, yellow, and red, respectively. Laser Power: Low; PMT: 5%.
